# Random Field Ising Model Criticality in a Complex Binary Liquid System

**DOI:** 10.3390/nano14131125

**Published:** 2024-06-29

**Authors:** Henrich Frielinghaus, Purushottam S. Dubey, Debasish Saha, Eunjoo Shin, Olaf Holderer, Jan V. Sengers, Stephan Förster

**Affiliations:** 1 Forschungszentrum Jülich GmbH, Jülich Center for Neutron Scattering JCNS-4 at MLZ, Lichtenbergstrasse 1, 85747 Garching, Germany; 2 Korea Atomic Energy Research Institute, 111, Daedeok-daero 989beon-gil, Yuseong-gu, Daejeon 34057, Republic of Korea; 3 Institute for Physical Science and Technology, University of Maryland, College Park, MD 20742, USA; 4 Forschungszentrum Jülich GmbH, Jülich Center for Neutron Scattering JCNS-1, Leo-Brandt Str., 52425 Jülich, Germany

**Keywords:** critical fluctuations, critical exponents, SANS, confinement, porous aerogel, random-field Ising model

## Abstract

While Ising criticality in classical liquids has been firmly established both theoretically and experimentally, much less is known about criticality in liquids in which the growth of the correlation length is frustrated by finite-size effects. A theoretical approach for dealing with this issue is the random-field Ising model (RFIM). While experimental critical-exponent values have been reported for magnetic samples (here, we consider γ, ν and η), little experimental information is available for critical fluctuations in corresponding liquid systems. In this paper, we present a study on a binary liquid consisting of 3-methyl pyridine and heavy water in a very light-weight porous gel. We find that the experimental results are in agreement with the theoretical predictions from the RFIM.

## 1. Introduction

Complex fluids have a wide range of applications in industry. Some examples are micro- or nano-reactors for chemistry [1], controlled drug release [2], enhanced oil recovery [3], electrolytes in batteries [4] and fuel or electrolyzer cells [5], many of which deal with multiphase coexistence and face issues of miscibility. In microemulsions with two phases, the production of nanoparticles can be controlled in terms of size and shape [1]. For pharmaceutical applications, the formation of small compartments that crystallize may be of major importance for encapsulation and controlled release [2]. In surfactant flooding, the surfactant fluid is purposely formulated with respect to interfacial tension, wettability alteration, foam generation and emulsification [3]. Often, the viscosity of the micellar suspension is extremely important too. Polar and less polar (e.g., ethylene carbonate) substances are also important as electrolytes for batteries [4]. They support higher voltages in applications where the electrolyte passivates the aggressive electrodes through the cathode electrolyte interphase and/or the solid electrolyte interphase. Also, hydrophobic fluorinated organic molecules can support this process while being less flammable. However, they are not the best environmental solution. Fuel and electrolyzer cells frequently use proton-conducting liquids—often with a polymeric scaffold for mechanical stability [5]. The electrolyte can be phosphoric acid, potassium hydroxide or other alkalines with smaller amounts of water. All these examples deal with hydrophilic/hydrophobic or amphiphilic mixtures and introduce a certain kind of randomness to the structure.

One way of obtaining some insight into this topic may be by studying the critical behavior of binary liquids in porous media. In the absence of surface effects, binary liquids display 3-dimensional (3D, d=3) Ising criticality [6,7,8,9,10,11]. However, for binary liquids in porous media, there are possible changes in their critical behavior [12,13,14]. While supercritical CO_2_ is not a binary system, in this case, the correlation length in CO_2_ near the critical point has been shown to be limited by the pore size of an aerogel [15]. Binary mixtures of 2,6-lutidine and D_2_O have been studied with small-angle neutron-scattering (SANS) experiments [16,17]. Here, the emphasis was on the theoretical understanding of the scattering functions themselves. The correlation length could grow rather large compared to the pore size, although it is also limited by the pores. A conclusion on the nature of the observed critical behavior has not been obtained. A textbook dedicated to criticality in systems in porous media has been provided by Melnichenko [18].

Concerning critical exponents, the random-field Ising model (RFIM) is an important model that may describe large correlation lengths compared to smaller structures of randomness [19]. Critical exponents have been listed from several theoretical approaches [20,21,22,23,24]. Most experimental values have been reported for magnetic samples, primarily dilute antiferromagnets [25]. However, experimental evidence for the presence of RFIM criticality in liquid systems is largely absent. One measurement of one exponent (β) has been reported for N_2_ in an aerogel [26].

In this paper, we report a detailed experimental study on the critical behavior of a binary liquid in a highly porous aerogel. The liquids were 3-methyl pyridine (3MP) and heavy water (D_2_O). In the literature, this system has been confirmed as an ideal 3D Ising system without aerogel [27,28]. But by adding antagonistic salt or surfactants, the third component locally imposes a lamellar order (also called charge density waves) that confines the two main components to two dimensions. More details about this different topic can be found in the literature [27,28,29].

We first summarize existing theoretical concepts. Then, we describe SANS curves using the small-angle scattering model and define the necessary parameters. Finally, we shall discuss the critical exponents deduced from the analysis of the experimental data and shall try to reconcile them with the literature and the available theoretical concepts.

## 2. Theory

In this section, we summarize the ideas of the best developed scattering theories that have successfully been applied to experimental data [15,16,17]. From this, we further discuss the criticality of selected parameters that do depend on temperature. The scattering function applied in analyzing our SANS measurements is similar to the expression of Sinha [16]:

(1) $$\frac{d\Sigma }{d\Omega }\left(Q\right)=\frac{I_{\mathrm{OZ},\mathrm{mod}}\left(0\right)}{{\left(1+\xi^{2}Q^{2}\right)}^{1-\eta /2}}+\frac{I_{\mathrm{RF}}\left(0\right)}{{\left(1+\xi^{2}Q^{2}\right)}^{2}}\cdot S_{G}\left(Q\right)$$

The macroscopic cross section of the SANS experiment dΣ/dΩ is normalized to absolute units and depends isotropically on the scattering wave number *Q*. The first term is based on the classical Ornstein–Zernike expression [30,31], but modified by Fisher [30] in order to obtain the large-*Q* scaling corrected by the critical correlation function exponent η. This modified Ornstein–Zernike expression contains an amplitude $I_{\mathrm{OZ},\mathrm{mod}}\left(0\right)$ and the correlation length ξ for the critical fluid. The next term is based on the response function and involves the square of the ideal Lorentzian of the Ornstein–Zernike expression. Again, we have an amplitude $I_{\mathrm{RF}}\left(0\right)$. The original theory [16] contains an additional constant. However, we estimate this constant to be approx. 2% or less compared to the square root of the amplitude $I_{\mathrm{RF}}\left(0\right)$. Hence, we neglected this additional term as was done by Melnichenko [15]. The last term in Equation (1) contains the structure factor $S_{G}$ of the aerogel. It was introduced by Sinha and Melnichenko [15,16]:

(2) $$S_{G}\left(Q\right)=\frac{\sin[\left(D_{f}-1\right)\arctan\left(\xi_{G}Q\right)]}{\left(D_{f}-1\right)\xi_{G}Q\cdot {\left(1+\xi_{G}^{2}Q^{2}\right)}^{(D_{f}-1)/2}}$$

Here, we have the correlation length of the aerogel $\xi_{G}$ and its fractal dimension $D_{f}$. Both terms are material parameters and do not depend on temperature. The structure factor $S_{G}$ is normalized such that with a limit of small *Q*, it approaches unity. The whole theory is applicable when the sizes $\xi_{G}>R_{P}>\xi$ are ordered. As we will see below, $\xi_{G}=9.8$ nm, and the pore size $R_{P}=3$ to 3.5 nm. The correlation length ξ of the liquid is lower at the lowest temperature, but can exceed $R_{P}$ at higher temperatures (see discussion of ξ). As we will see, in the latter case, the full theory cannot be applied over the full *Q*-range anymore.

Near the critical point, the temperature dependence of the amplitude $I_{\mathrm{OZ},\mathrm{mod}}$(0) of the Ornstein–Zernike term and of the correlation length in Equation (1) are represented by power laws of the form

(3) $$I_{\mathrm{OZ},\mathrm{mod}}\left(0\right)=I_{0}\cdot \tau^{-\gamma }$$

and

(4) $$\xi =\xi_{0}\cdot \tau^{-\nu }$$

Here, $\tau =|1-T/T_{c}|$ in terms of the temperature *T* and the critical temperature $T_{c}$. For the amplitude of the response function, we assume a similar scaling as for the Ornstein–Zernike term. So far, this is not strictly supported by theories, but in our study, it seems to be confirmed empirically (see discussion of amplitudes $I_{0}$ and $I_{\mathrm{RF},0}$):

(5) $$I_{\mathrm{RF}}\left(0\right)=I_{\mathrm{RF},0}\cdot \tau^{-\gamma_{\mathrm{RF}}}$$

Values for the critical exponents γ, ν and η are presented in Table 1. We quote values for the ideal 3-dimensional Ising behavior and include the ones for the random-field Ising model. For the latter, we summarize theoretical and experimental values (or ranges). While there exists agreement for the ideal Ising exponents with minor uncertainties, the theories for the RFIM are distributed depending on detailed assumptions of the model. The experimental data in the literature have only been obtained for magnetic samples. To our knowledge, we have, for the first time, obtained similar exponent values for a liquid system.

The whole set of experimental parameters that were experimentally obtained from SANS experiments are summarized in Table A1 in Appendix A.

## 3. Materials and Experiments

3-methyl-pyridine (3MP, 99.5% purity) was purchased from Sigma Aldrich, Taufkirchen, and used as received. Heavy water (D_2_O, 99.8% purity) was purchased from Armar Chemicals, Döttingen, and used as received. The aerogel was purchased from Stadur-Süd, Pliezhausen, with a density of 0.07 g/cm^3^. The flakes were filled in with a spatula to a banjo Hellma cell (1 mm thickness) and gently compressed before the mixed fluid was added. Information about the SANS experimental facility used in this work was already presented in a previous publication [29]. The 3MP/D_2_O mixtures were mixed by volume (35%vol 3MP). The mixture displays a lower critical solution temperature [29] of 36.8 °C as a binary system and of 44 °C in the aerogel. This indicates that the liquids are in a one-phase state. The scattering length densities of D_2_O/3MP/aerogel are (6.36/1.43/(3.48 ± 0.1)) × 10^−4^ nm^2^. This finding states that the liquid–liquid contrast is approx. 50 times stronger than the liquid–solid contrast.

## 4. Analysis of the Experimental Data

**Figure 1:**
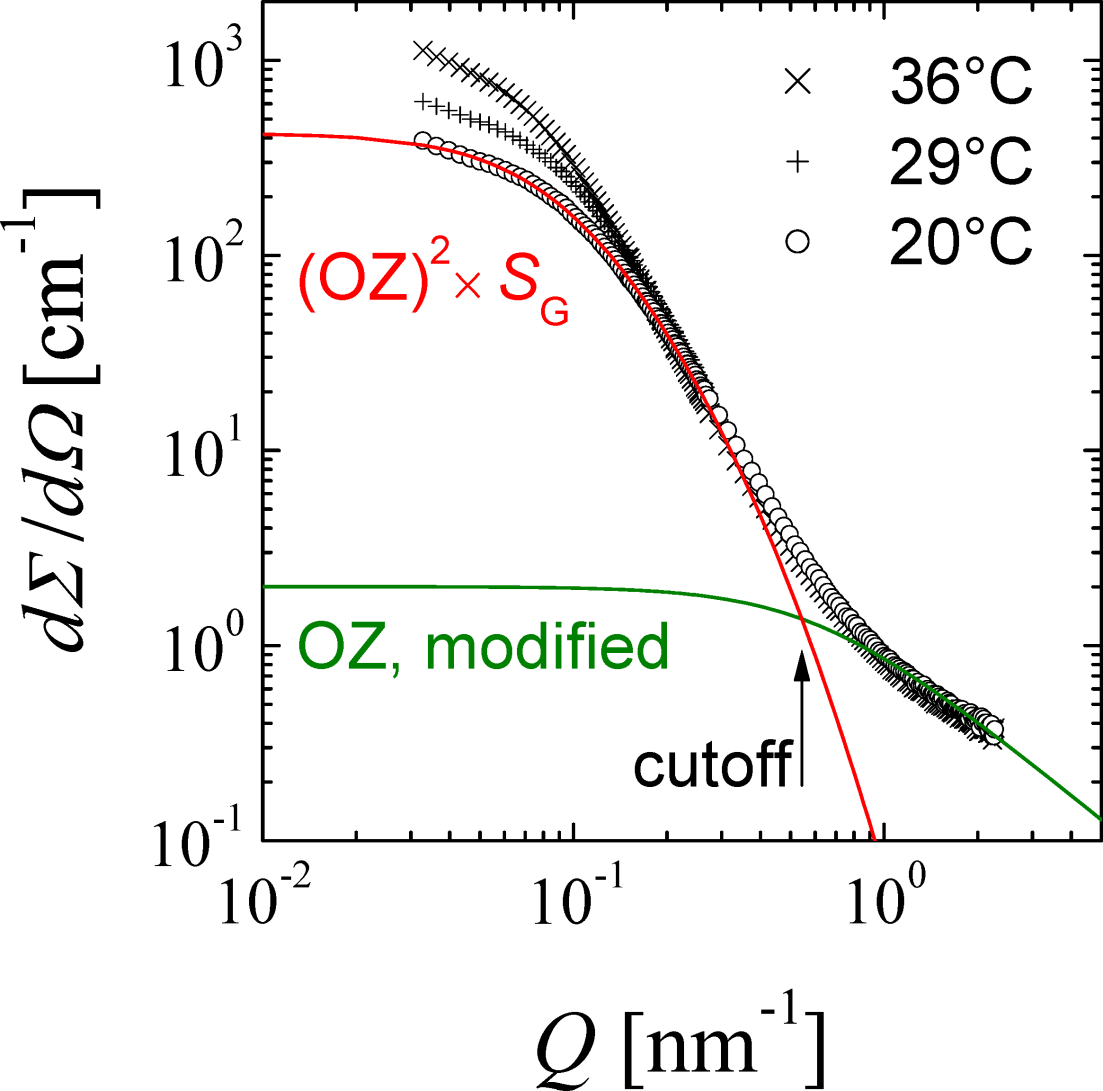
The macroscopic cross section as a function of the scattering wave number for different temperatures. The lines (green and red) indicate the separated contributions of the modified Ornstein–Zernike expression and the response function (first and second term in Equation (1). The statistical errors are of the size of the symbols or smaller.

The SANS experiments were conducted with a set of different temperatures ranging from 20 °C to 36 °C. A selection of three different data sets are depicted in Figure 1 in a log–log scale. We can see monotonically decaying intensities toward higher *Q*. For the lowest temperature, we applied the full theory of Equation (1). The two different contributions in Equation (1) are represented by separate curves. We have the modified Ornstein–Zernike expression dominating for Q>0.55 nm^−1^. At the highest *Q*, the power law is connected to the exponent η=0.7±0.1. At lower *Q*, the fractal structure of the aerogel $S_{G}$ is dominating. The square of the Lorentzian (or Ornstein–Zernike) has two influences: (a) it provides a cut-off for the glass structure and (b) it modifies the Gunier scattering at the lowest *Q*, i.e., the initial decay.

When we look at the curves for higher temperatures, we see that the power law at the highest *Q* does not change considerably. This means that the exact amplitude of this term may not be well determined by model fitting. Second, the cut-off of the fractal glass structure does not change, while the low-*Q* decay does change. In theory (Equation (1)), both features would be affected by the correlation length ξ simultaneously. So the original theory contradicts the experimental findings, and we have to apply a different approach for the description of the data.

First, we applied the original theory to the lowest temperature and assumed that the cut-off and low-*Q* decrease are well coupled. We obtain the correct amplitude and correlation length of the modified Ornstein–Zernike term, and simultaneously also fit the fractal glass structure with its correlation length $\xi_{G}=\left(9.8\pm 0.2\right)$ nm and dimension $D_{f}=2.44\pm 0.03$. The glass structure agrees very well with the study of Melnichenko [15], and so we assume that the given pore radius $R_{P}=3$ to 3.5 nm also applies to our study. The coincidence $R_{P}\approx Q_{\mathrm{cutoff}}^{-1}$ should be noted. For the other curves, we only considered the lower *Q*-end (Q<0.21 nm^−1^) and fitted the fluid correlation length ξ, while the glass structure factor (Equation (2)) was kept constant. The overall amplitude $I_{\mathrm{RF}}\left(0\right)$ was obtained from the fit as well.

**Figure 2:**
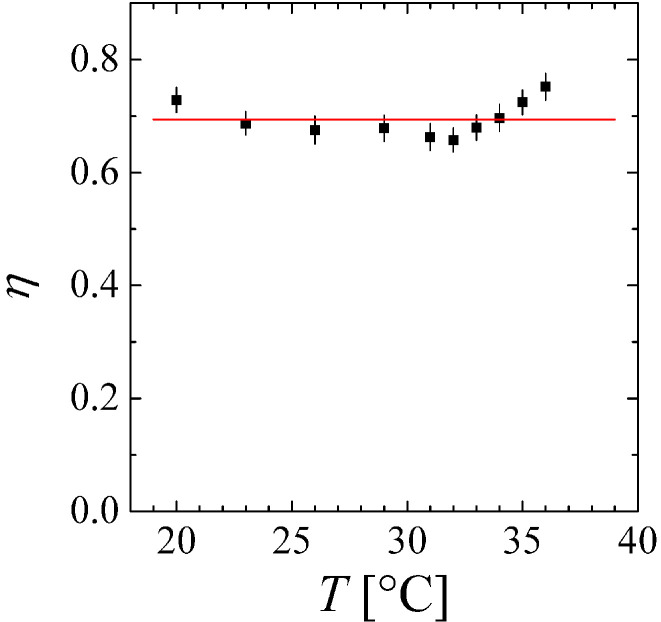
The critical correlation function exponent η as a function of temperature. Within the error bars, we conclude that η is a constant indeed.

We then focused separately on the high-*Q* end of the SANS curves (Q>0.85 nm^−1^) and fitted the Ornstein–Zernike expression (Equation (1)) only with a free exponent η for all temperatures. The collected values are depicted in Figure 2. The temperature dependence is negligible within the given errors. So, we obtain a constant η=0.69±0.01, which is much more precise than from fitting of the full theory (Equation (1)) over the full *Q*-range for only one temperature.

**Figure 3:**
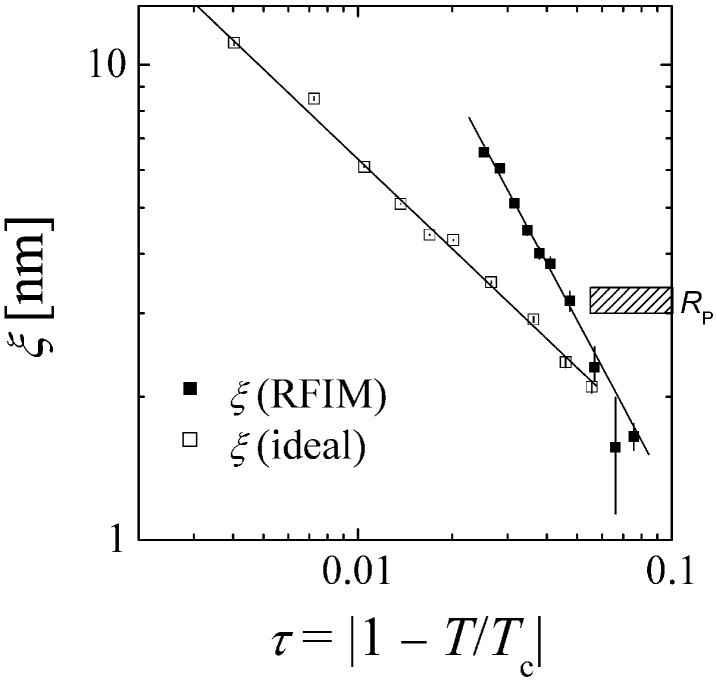
The correlation length as a function of the reduced temperature. The solid symbols indicate the experimental data for the liquid system with aerogel (RFIM), and the open symbols indicate the experimental data obtained earlier for the liquid system without aerogel (ideal) satisfying ideal Ising criticality [29]. The lines indicate the critical scaling (Equation (4) with weighted errors). The errors bars are all shown and may be smaller than the size of the symbols if invisible.

The critical behavior of ξ for our binary liquid in an aerogel is compared to the one for the same binary liquid in the absence of an aerogel, which satisfies ideal Ising behavior [29], shown in Figure 3.

We also indicate the size of the pore $R_{P}$, where a transition of the behavior for our confined system could be expected. Nevertheless, a perfect power law behavior is observed with a critical exponent of ν=1.24±0.05. For most of the temperatures, the ξ of the ideal Ising system lies below, and the critical exponent is considerably lower (ν=0.63). So, the stronger growth of ξ in our liquid in aerogel indicates less interactions of domains with neighboring domains, similar to the ideal case of lower dimensionality [29,32]. The compartments of single pores are slightly isolated from neighboring pores.

**Figure 4:**
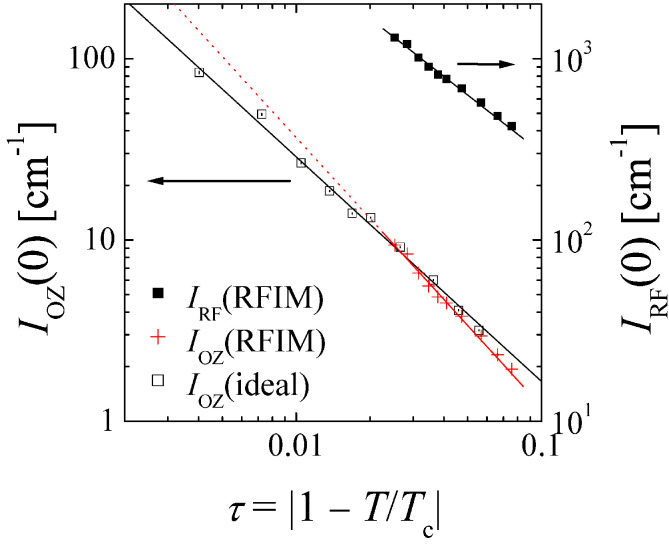
The forward scattering as a function of the reduced temperature from the two different terms of Equation (1) (crosses and solid symbols) compared to data obtained earlier (open symbols) for the liquid system without aerogel (ideal) satisfying ideal Ising criticality [29]. The lines indicate the critical scaling (Equations (3) and (5) with weighted errors). The errors bars are all shown and may be smaller than the size of the symbols if invisible.

The criticality of the amplitude $I_{\mathrm{RF}}\left(0\right)$ is depicted in Figure 4. Again, we see a very simple power law behavior with a critical exponent $\gamma_{\mathrm{RF}}=1.06\pm 0.05$. From the study by Sinha [16], we infer the scaling between the two amplitudes $I_{\mathrm{RF}}\left(0\right)$ and $I_{\mathrm{OZ},\mathrm{mod}}\left(0\right)$ and apply it to our data to obtain a good guess for $I_{\mathrm{OZ},\mathrm{mod}}\left(0\right)$. They are similar to those found for the ideal Ising mixture. However, the critical exponents of the binary liquid in aerogel differ ($\gamma =\gamma_{\mathrm{RF}}/\left(2\cdot 0.358\right)=1.48\pm 0.15$) (see also Appendix A) substantially from those for the ideal Ising liquid mixture (γ=1.24). This difference would be dominating much closer to the critical temperature. Again, this would also indicate less interactions between neighboring domains, just less pronounced here.

We stress here that in either case of the critical behavior (ξ and $I_{\mathrm{RF}}\left(0\right)$), there is no indication of a change when ξ is crossing the pore size $R_{P}$. This indicates that our binary liquid is only weakly interacting with the aerogel, i.e., there seems to not be a strong preference of either component to the glass. Then, the correlation length ξ may grow unhindered, and it may exceed the pore size $R_{P}$. However, there is a tendency to fill pores quite completely, and the interactions between neighboring pores are reduced [14], similar to the ideal case of lower dimensionality (2 dimensions: γ=7/4, ν=1 and η=1/4 [33]). This is expressed by larger critical exponents and the larger values of ξ and $I_{\mathrm{RF}}\left(0\right)$ when approaching the critical temperature. The much larger exponent η indicates sharper interfaces between the domains compared to the ideal Ising behavior.

When we consider the connections of the three critical exponents, γ, ν and η, that we determined independently, there are theoretical scaling relations that could help to verify the validity of our measurements. The classical relation γ/ν=2−η [34] holds well within the experimental errors. But also, the hyperscaling relation γ/ν=d/2−β [34] seems to be only slightly off when we assume that ±0≲β≲0.35 [20,26]. So, we would not really prefer one of the two scaling relations.

Lastly, we discuss the shift of the critical temperature from the ideal Ising liquid mixture (*T*_c_ = 36.8 °C) to our liquid mixture in an aerogel (*T*_c_ = 44 °C). So, the one phase region is extended by exposing the fluid to the aerogel. This is similar to adding ions to the fluid [35]. Thus, the two disturbances seem to act similarly here.

## 5. Summary

We presented an analysis of our SANS curves obtained for the binary 3MP/D_2_O fluid in a light-weight porous aerogel as a function of temperature. From the data at high scattering wave numbers *Q*, we obtained the critical correlation function exponent η. We also determined the critical behavior of the correlation length ξ and the amplitudes of the Ornstein–Zernike scattering and the response function, i.e., $I_{\mathrm{OZ},\mathrm{mod}}\left(0\right)$ and $I_{\mathrm{RF}}\left(0\right)$, yielding the critical exponents ν and γ. We found that the critical exponent values for our binary liquid system, as well as the scaling relations, are in substatial agreement with values reported in the literature for the RFIM. Thus, we conclude that our fluid in the aerogel displays the criticality of the RFIM. The trend toward higher values of the exponents is to be seen in parallel to lower dimensionalities because the coordination number of the pores is reduced with respect to ideally free domains. The RFIM-like critical exponent values found from our experiments are the first ones of this kind obtained for liquid systems.

The constant cut-off between the Ornstein–Zernike and response function scattering, the very high amplitude $I_{\mathrm{RF}}\left(0\right)$ and the extremely high exponent η let us conclude that the pores in the aerogel are filled with quite pure components, and the interfaces between the domains are quite sharp. This finding might be interesting for the formulation of synthetic tissues for drug release, for the finding of membranes that skim fluids and for constructing scaffolds, i.e., membranes, for fuel and electrolyzer cells.

## Figures and Tables

**Table 1:** Summary of the critical exponents in three dimensions. The first column summarizes the theoretical values of the ideal Ising criticality [10,11]. The second column collects a spread of theoretical values for the RFIM [20,21,22,23,24]. The third column summarizes experimental exponent values found for magnetic systems [25].

	Ideal	Theory	Experiment	This
Exponent	Ising	RFIM	RFIM	Study
γ	1.238 ± 0.012	1.5–2.0	1.75 (±0.25)	1.48 ± 0.15
ν	0.629 ± 0.003	1.0–1.4	1.1 (±0.2)	1.24 ± 0.05
η	0.032 ± 0.013	0.25–0.52	0.16–0.5 (–)	0.69 ± 0.01

**Figure A1:**
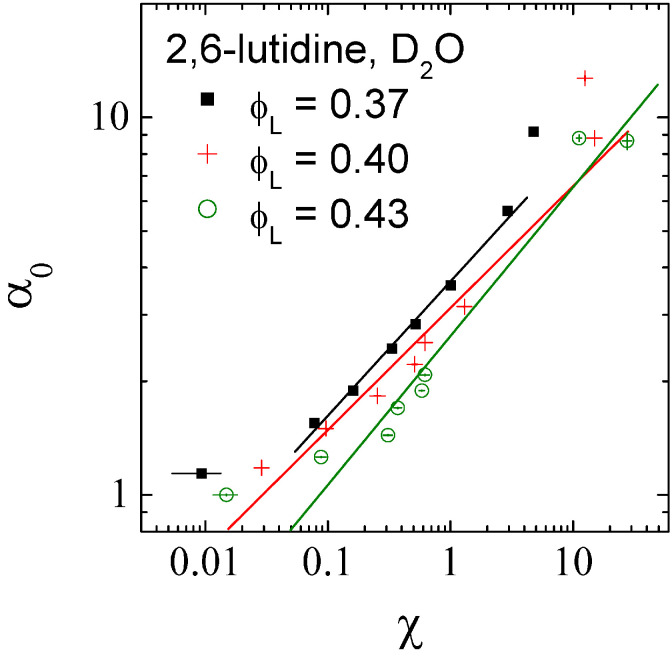
The amplitude of the response function $\alpha_{0}$ as a function of the Ornstein–Zernicke amplitude χ from the experiments of Sinha [16] for the system of 2,6-lutidine/D_2_O mixture in porous vycor glass. An apparent average slope of 0.36 was determined. Error bars appear within the symbols.

## Data Availability

Data are contained within the article.

## Appendix A. Scaling of the Amplitudes *I*_OZ_(0) and *I*_RF_(0)

In this work, we only were able to extract the temperature dependence of the amplitude $I_{\mathrm{RF}}\left(0\right)$ for the response function, while the amplitude $I_{\mathrm{OZ}}\left(0\right)$ was hidden below the much stronger response function term. Additionally, the nearly constant cutoff of the response function term did not allow for the extrapolation of the high-*Q* scattering to a forward scattering Q→0. So, we tried to obtain an estimate of the Ornstein–Zernicke amplitude $I_{\mathrm{OZ}}\left(0\right)$ using the empirical scaling that Sinha obtained in his experimental study [16]. We collected his amplitudes of the response function $\alpha_{0}$ ($\alpha_{0}^{2}∼I_{\mathrm{RF}}\left(0\right)$) as a function of the amplitude and the Ornstein–Zernicke term $\chi ∼I_{\mathrm{OZ}}\left(0\right)$ and obtained a scaling relation with an exponent ϵ (Figure A1). For his data sets with different concentrations $\phi_{L}$, we obtained an average ϵ=0.358±0.020 for the scaling $\alpha_{0}={\hat{\alpha }}_{0}\chi^{\epsilon }$. We just have to stress that his scaling was obtained for a 2,6-lutidine/D_2_O mixture in a porous vycor glass. However, we believe that the scaling exponent ϵ should be rather universal.

**Table A1:** Summary of the parameters determined experimentally from SANS experiments.

Parameter in Aerogel	Value	Equation	Figure
$D_{f}$	2.44 ± 0.03	(2)	Figure 1
$\xi_{G}$	9.8 ± 0.2 nm	(2)	Figure 1
η	0.7 ± 0.1	(1)	Figure 1
	0.69 ± 0.01	cst.	Figure 2
γ	1.48 ± 0.15	(3)	Figure 4
ν	1.24 ± 0.05	(4)	Figure 3
$\gamma_{\mathrm{RF}}$	1.06 ± 0.05	(5)	Figure 4
$I_{0}$	0.040 ± 0.009 cm^−1^	(3)	Figure 4
$\xi_{0}$	0.071 ± 0.013 nm	(4)	Figure 3
$I_{\mathrm{RF},0}$	18.3 ± 2.8 cm^−1^	(5)	Figure 4
$T_{C}$	44 ± 1 °C	–	–
in bulk			reference
η	0.04 ± 0.04	–	[32]
γ	1.239 fixed	(3)	[29]
ν	0.629 fixed	(4)	[29]
$I_{0}$	0.088 ± 0.003 cm^−1^	(Figure 4)	[29]
$\xi_{0}$	1.15 ± 0.03 nm	(Figure 3)	[29]
$T_{C}$	36.8 ± 0.3 °C	–	[29]
